# Identifying Neurobehavioral Biomarkers of Anxiety and Treatment Response Using Virtual Reality, Electroencephalography, Magnetic Resonance Imaging, and Related Multimodal Assessments: A Longitudinal Study Protocol

**DOI:** 10.3390/jcm15010007

**Published:** 2025-12-19

**Authors:** Hyemin Oh, Jiook Cha, Byung-Hoon Kim, Kang-Seob Oh, Young Chul Shin, Sang-Won Jeon, Sung Joon Cho, Junhyung Kim

**Affiliations:** 1 Department of Psychiatry, Kangbuk Samsung Hospital, Sungkyunkwan University School of Medicine, 29 Saemunan-ro, Jongno-gu, Seoul 03181, Republic of Korea; 2 Department of Brain and Cognitive Sciences, Seoul National University, 1 Gwanak-ro, Gwanak-gu, Seoul 08826, Republic of Korea; 3 Interdisciplinary Program in Artificial Intelligence, Seoul National University, 1 Gwanak-ro, Gwanak-gu, Seoul 08826, Republic of Korea; 4 Department of Psychology, Seoul National University, 1 Gwanak-ro, Gwanak-gu, Seoul 08826, Republic of Korea; 5 Institute of Psychological Science, Seoul National University, 1 Gwanak-ro, Gwanak-gu, Seoul 08826, Republic of Korea; 6 Department of Psychiatry, Yonsei University College of Medicine, 50-1 Yonsei-ro, Seodaemun-gu, Seoul 03722, Republic of Korea; 7 Institute of Behavioral Sciences in Medicine, Yonsei University College of Medicine, 50-1 Yonsei-ro, Seodaemun-gu, Seoul 03722, Republic of Korea; 8 Workplace Mental Health Institute, Kangbuk Samsung Hospital, Sungkyunkwan University School of Medicine, 29 Saemunan-ro, Jongno-gu, Seoul 03181, Republic of Korea

**Keywords:** anxiety disorders, virtual reality, electroencephalography, magnetic resonance imaging, biomarker

## Abstract

**Background/Objectives**: Anxiety disorders are highly prevalent and impairing psychiatric conditions. Conventional diagnostic approaches based on symptom checklists lack biological specificity and often fail to guide treatment decisions effectively. This study protocol outlines a multidimensional, prospective investigation designed to identify behavioral and neurobiological biomarkers predictive of treatment response in individuals with anxiety-related symptoms, grounded in the Research Domain Criteria framework. **Methods**: This observational, longitudinal study (NCT06773585) will include a transdiagnostic sample of clinical anxiety group alongside a healthy control group (185 participants, including 145 patients with anxiety disorders and 40 healthy controls). Participants will undergo comprehensive baseline assessments, including clinical interviews, self-report questionnaires, a virtual reality (VR)-based behavioral task, electroencephalography (EEG), electrocardiography (ECG), and structural and functional brain magnetic resonance imaging. Follow-up assessments will be conducted at 2, 6, and 12 months, with recruitment and data collection planned from 2024 to 2029. These complementary modalities are integrated to capture behavioral, physiological, and neural indicators of anxiety and its treatment response. Multimodal baseline features will be used to construct machine-learning models predicting treatment response, defined as ≥40% reduction in anxiety severity scores. Longitudinal analyses will examine symptom trajectories and neural mechanisms associated with response. Neurobiological comparisons will be made across timepoints and between responders, non-responders, and healthy controls. **Conclusions**: By identifying objective, biologically grounded markers of anxiety and treatment response, our findings will contribute to the development of personalized assessment tools and scalable digital interventions for psychiatric care.

## 1. Introduction

Anxiety disorders are among the most prevalent and disabling psychiatric conditions, affecting psychological functioning, interpersonal relationships, and overall life satisfaction worldwide [1]. Recent epidemiological trends indicate a global rise in the incidence of anxiety disorders, paralleled by increasing healthcare costs and socioeconomic burdens [2]. Despite their high prevalence, anxiety disorders remain biologically elusive.

Recent large-scale genome-wide association studies have revealed a highly polygenic architecture underlying anxiety and related affective traits, identifying numerous genome-wide significant loci across international biobank and consortium datasets [3,4,5]. However, the proportion of variance explained by individual variants remains small, and mechanistically interpretable or pharmacologically actionable targets are still scarce. These findings highlight the need to integrate genomic insights with multimodal neurobehavioral data to elucidate how genetic risk translates into clinical expression and therapeutic responsiveness [3]. Furthermore, treatment outcomes remain difficult to predict, as current diagnostic systems provide limited guidance in identifying individuals likely to benefit from specific interventions [6,7]. These challenges underscore the need for a more biologically grounded, individualized approach to the assessment and management of anxiety.

Traditional psychiatric classification systems, such as the Diagnostic and Statistical Manual of Mental Disorders (DSM), rely primarily on symptom-based categories and clinical interviews. These approaches, while valuable for diagnostic consensus, are inherently limited by subjectivity, poor inter-rater reliability, and a lack of neurobiological specificity [8,9]. The categorical nature of DSM-based diagnoses obscures underlying heterogeneity and contributes to substantial overlap across disorders, thereby hindering biomarker discovery and the development of precision-guided treatments [10].

In response to these limitations, the US National Institute of Mental Health proposed the Research Domain Criteria (RDoC) framework as an alternative dimensional model of psychopathology. RDoC promotes the integration of data across multiple units of analysis—including genetics, circuits, behavior, and self-report—within transdiagnostic constructs. Within this framework, anxiety is conceptualized primarily under the Negative Valence Systems domain, particularly the “Potential Threat” construct [11].

The RDoC approach has demonstrated substantial empirical progress. Large-scale initiatives and meta-analytic efforts have established reproducible neural and behavioral constructs across affective and anxiety spectra [12,13]. For instance, recent RDoC-aligned neuroimaging and electrophysiological studies showed a shared threat-anticipation circuit involving the central extended amygdala, periaqueductal gray, and prefrontal cortex that is dynamically engaged during certain and uncertain threat [14]. Building on this foundational understanding of threat processing circuitry, subsequent studies have mapped dysregulation within this frontal-limbic network to heightened threat processing and impaired emotion regulation across generalized anxiety disorder (GAD) and post-traumatic stress disorder [15]. These advances illustrate how integrating neural-circuit, behavioral, and self-report measures under transdiagnostic constructs can reveal common mechanisms underlying anxiety-related phenomena [16,17,18]. These findings suggest that brain circuits involved in threat detection and regulation may serve as transdiagnostic, mechanistic biomarkers of anxiety. Nevertheless, although some longitudinal and multimodal studies have examined neurobiological correlations of anxiety, relatively few have focused on prospective prediction of treatment response using integrated VR-based behavioral and neurobiological assessments within an RDoC-aligned framework. To address this gap, we proposed a longitudinal, multimodal approach—combining virtual reality (VR), electroencephalography (EEG), and magnetic resonance imaging (MRI)—to address the gap by offering a more comprehensive, predictive model of anxiety.

Empirical evidence from individual modalities has also advanced our understanding of anxiety-related processes. EEG studies have identified abnormal theta and beta band activity and altered error-related negativity during anticipatory and conflict processing in anxiety [19,20]. Electrocardiography (ECG) has consistently shown reduced heart-rate variability and increased sympathetic tone, reflecting heightened physiological arousal and impaired stress regulation [21,22]. Neuroimaging studies further demonstrate hyperactivation of the amygdala, insula, and prefrontal regions, along with disrupted connectivity within the default mode and salience networks in anxiety disorders [23,24].

VR offers a promising platform for implementing RDoC-informed assessments by enabling standardized, ecologically valid exposure to anxiety-provoking stimuli [25]. VR environments can be integrated with real-time biosignal monitoring—including EEG, ECG, and 3D skeletal tracking—to capture multimodal, task-evoked responses [26]. This capacity allows for the measurement of behavioral, physiological, and neural responses within a controlled but naturalistic context. Conceptually, this multimodal assessment approach maps directly onto RDoC domains such as Negative Valence and Arousal/Regulatory Systems and offers scalability, reproducibility, and objectivity that are difficult to achieve with traditional self-report methods.

Considering these opportunities, we developed a prospective observational study aimed at identifying neurobehavioral biomarkers of anxiety and predicting treatment response. Although participants are recruited based on DSM-5 diagnoses of anxiety disorders—including panic disorder, social anxiety disorder (SAD), GAD, agoraphobia, and specific phobia—the analytic focus is on dimensional variations in anxiety symptoms that cut across diagnostic boundaries. This transdiagnostic strategy acknowledges that current categorical systems provide limited information about therapeutic responsiveness and seeks to characterize symptom-level mechanisms that underlie anxiety across disorders. The study integrates VR-based behavioral tasks, physiological monitoring, and multimodal neuroimaging within an RDoC-aligned conceptual framework. By combining these multimodal data with conventional clinical assessments, it aims to contribute to the development of biologically informed, scalable tools for personalized psychiatric care.

The primary objective of this study is to delineate neurobehavioral biomarkers associated with treatment response in individuals with anxiety-related disorders using a transdiagnostic approach. Specifically, the present study aims to delineate patterns of neural and behavioral markers that cut across diagnostic boundaries—including panic disorder, SAD, GAD, agoraphobia, and specific phobia—thereby capturing shared pathophysiological processes relevant to therapeutic responsiveness. Healthy controls are included as a reference group to distinguish disorder-related changes from normative variability. We hypothesized that multimodal integration of VR, EEG, 3D skeletal tracking, and neuroimaging data will enable a more mechanistic and predictive understanding of anxiety and its treatment response beyond categorical diagnoses.

## 2. Materials and Methods

### 2.1. Design

This is a prospective, observational investigation designed to identify factors associated with treatment response and prognosis in a clinical anxiety group. The study population comprises two groups: individuals with anxiety disorders and a healthy control group. The anxiety group will complete assessments at baseline, 4 months, and 12 months, whereas the control group will undergo a single baseline assessment. The overall follow-up period is 12 months, with participant enrollment and data collection planned from 2024 through 2029.

**Figure 1:**
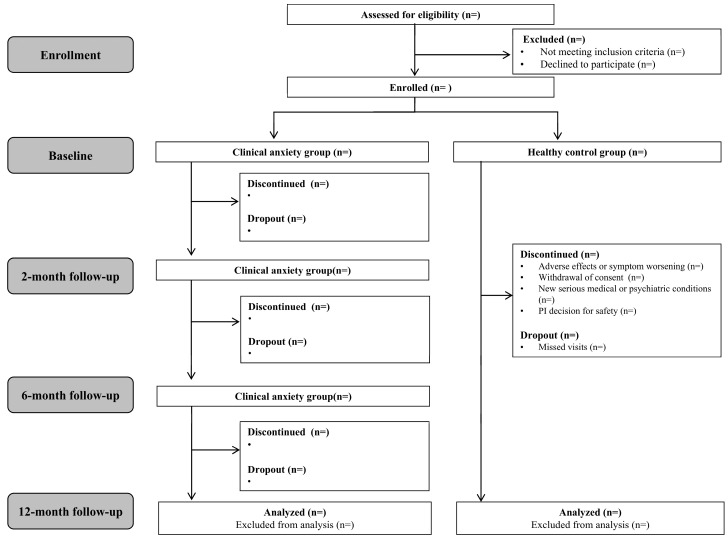
Study flowchart showing enrollment, allocation, follow-up, and analysis for both the clinical anxiety group and healthy control group. Participants in the clinical anxiety group received treatment-as-usual (e.g., pharmacotherapy or psychotherapy) as prescribed by their clinicians. Treatment response was defined as a ≥40% reduction in Panic Score Severity Scale (PDSS) scores at follow-up. No intervention was administered by the study team.

Although no structured therapeutic intervention is administered as part of the research protocol, participants in the clinical anxiety group may receive naturalistic treatment-as-usual, including pharmacological or psychotherapeutic interventions, as determined by their treating clinicians. Accordingly, the study does not involve randomization, allocation concealment, or blinding procedures. The overall design and temporal structure of the study assessments are illustrated in Figure 1.

### 2.2. Eligibility Criteria

Participants will be assigned to either the clinical anxiety group or the healthy control group according to the following inclusion and exclusion criteria:

#### 2.2.1. Inclusion Criteria

The inclusion criteria for the clinical anxiety group are (1) adults aged between 19 and 60 years; (2) currently diagnosed with an anxiety disorder based on DSM-5 criteria, specifically including panic disorder, SAD, GAD, agoraphobia, or specific phobia, confirmed by a board-certified psychiatrist (individuals who have been previously treated for anxiety are not included); (3) ability to understand the purpose and procedures of the study and provide written informed consent, and (4) no history of major neurological disorders or psychotic illness.

The inclusion criteria for the healthy control group are (1) adults aged between 19 and 60 years; (2) no current or past psychiatric diagnoses and not currently using any psychotropic medication; and (3) ability to fully understand the purpose and procedures of the study and provide written informed consent.

#### 2.2.2. Exclusion Criteria

The exclusion criteria for the clinical anxiety group are (1) inability to read or understand the consent form; (2) significant difficulty using VR equipment (e.g., due to visual or vestibular impairment); (3) history of epilepsy, brain injury, or other neurological disorders; (4) severe physical illnesses (e.g., cancer, active tuberculosis, cardiovascular disease); (5) currently diagnosed with substance use disorder or alcohol use disorder (nicotine use will not be excluded); (6) presence of pacemakers or implanted metallic devices; (7) pregnancy or belonging to a legally or socially vulnerable population (e.g., institutionalized individuals or employees of the research facility); and (8) individuals who had previously completed a structured treatment protocol for anxiety within the past six months.

All exclusion criteria listed above apply to the healthy control group as well, with the addition of any previous or current psychiatric disorder.

### 2.3. Recruitment Process and Screening for Enrollment

The clinical anxiety group will be recruited through outpatient psychiatric clinics at Samsung Kangbuk Hospital, where attending psychiatrists will identify individuals who meet the inclusion criteria. The healthy control group will be recruited via public announcements on institutional bulletin boards.

To confirm eligibility and psychiatric status, all participants will be evaluated by a board-certified psychiatrist using structured diagnostic interviews. The Structured Clinical Interview for DSM-5 Disorders will be used to verify diagnoses in the clinical anxiety group [27], while the Mini International Neuropsychiatric Interview (MINI) 5.0.0 will be administered to screen for comorbid conditions or to confirm the absence of psychiatric history in healthy controls [28].

All eligible participants will receive a detailed explanation of the study’s purpose, procedures, potential risks, and their rights. Written informed consent will be obtained before any assessments are conducted. Participation is entirely voluntary, and individuals may withdraw at any time without affecting their clinical care.

### 2.4. Discontinuation and Dropout Criteria

Participants may be discontinued from the study under the following circumstances: emergence of adverse events or significant exacerbation of symptoms, voluntary withdrawal of consent, development of serious medical or psychiatric conditions that preclude continued participation, or the principal investigator’s judgment that termination is necessary for the participant’s well-being.

Dropout is defined as non-compliance with the study protocol, including missing scheduled assessments or failing to attend follow-up visits within the designated visit window (±15 days from the scheduled date).

In cases of either discontinuation or dropout, the principal investigator will inform the participant of the rationale and any necessary actions to be taken. The research team will take all reasonable steps to ensure participant safety, including offering follow-up care or appropriate referrals. With the participant’s consent, data collected prior to withdrawal may be retained and used for analysis.

### 2.5. Observational Parameters and Clinical and Laboratory Assessments

Participants in the clinical anxiety group will complete assessments at four timepoints: baseline and 2, 6, and 12 months. The healthy control group will be assessed at baseline and 12 months. The timepoints were chosen to efficiently assess symptom improvement at 2 months, check for relapse at 12 months for the clinical anxiety group, and confirm continued health for the healthy control group. These intervals also minimize participant burden while ensuring comprehensive data collection over the course of a year. Assessments will include clinical interviews, standardized self-report questionnaires, computerized neurocognitive testing, VR-based behavioral evaluations, physiological recordings (EEG and ECG), and multimodal brain imaging (structural, functional, and diffusion MRI).

Given the 12-month follow-up period and the multimodal nature of the assessments, challenges related to participant burden and attrition are anticipated. To mitigate these risks, the study incorporates flexible scheduling, abbreviated interim assessments at selected follow-up visits, and the option for remote administration of questionnaires where appropriate. These strategies are intended to support participant retention and ensure data completeness while maintaining methodological rigor.

**Figure 2:**
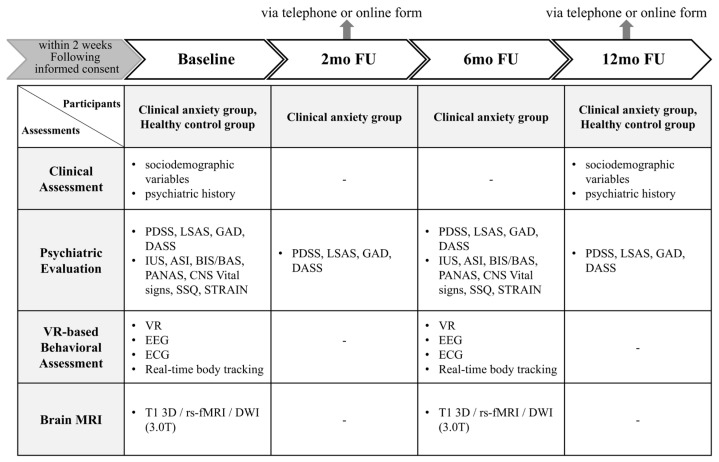
Schedule of assessments across study timepoints for the anxiety behavior and healthy control groups. Assessments include clinician-rated instruments, self-report questionnaires, neurocognitive testing, virtual reality (VR)-based behavioral tasks, physiological monitoring (EEG and ECG), and multimodal neuroimaging (structural MRI, resting-state functional MRI, and diffusion-weighted imaging). Assessments were selected based on their alignment with the Research Domain Criteria (RDoC) framework, targeting domains such as Negative Valence Systems, Cognitive Systems, and Arousal/Regulatory Systems. Interim visits (2 and 12 months) include abbreviated assessments to reduce participant burden. Remote administration may be used when appropriate. EEG, electroencephalography; ECG, electrocardiography; MRI, magnetic resonance imaging; PDSS, Panic Disorder Severity Scale; LSAS, Liebowitz Social Anxiety Scale; GAD-7, 7-item Generalized Anxiety Disorder Scale; DASS-21, Depression Anxiety Stress Scales-21; IUS, Intolerance of Uncertainty Scale; ASI-R, Anxiety Sensitivity Index-Revised; BIS/BAS, Behavioral Inhibition/Activation System Scales; PANAS, Positive and Negative Affect Schedule; SSQ, Simulator Sickness Questionnaire; STRAIN, Stress and Adversity Inventory; CNSVS, CNS Vital Signs Battery.

Figure 2 summarizes the timing and scope of these assessments across both groups. At each timepoint, specific instruments have been selected to capture transdiagnostic symptom dimensions and neurobehavioral mechanisms aligned with the RDoC framework—particularly domains related to Negative Valence Systems, Cognitive Systems, and Arousal/Regulatory Systems.

#### 2.5.1. Clinical Assessments

Clinical assessments will be conducted at the baseline and 12-month follow-up for all participants. Each session will be administered by a board-certified psychiatrist or trained research personnel. The assessment will include the collection of sociodemographic variables such as age, sex, education level, marital status, occupation, alcohol use, smoking status, height, and weight. Additionally, a structured evaluation of psychiatric history will be performed, including the onset and duration of anxiety symptoms, previous psychiatric diagnoses and treatments (treatment type, dosage, duration), and family history of psychiatric disorders.

These data will provide essential contextual information for interpreting behavioral, physiological, and neuroimaging findings, and for exploring clinical predictors of treatment response and prognosis. Some of these data will also be used as independent variables in the analysis.

#### 2.5.2. Evaluation of Anxiety Symptoms and Associated Psychiatric Conditions

To comprehensively assess anxiety and related cognitive-affective constructs, multiple validated self-report measures will be administered. Each instrument serves a distinct role within the analytic framework. Key clinician-rated and self-report instruments include the Panic Disorder Severity Scale (PDSS), the Depression Anxiety Stress Scales-21 (DASS-21), the Generalized Anxiety Disorder-7 (GAD-7), the Intolerance of Uncertainty Scale (IUS), and the Anxiety Sensitivity Index-Revised (ASI-R). Each instrument serves a distinct analytic role within the study framework. The PDSS functions as the primary measure of panic-related symptom severity and behavioral avoidance and is used to define treatment response. The DASS-21 Anxiety subscale serves as a secondary outcome reflecting generalized physiological and cognitive anxiety, while the GAD-7 provides a complementary index of generalized worry and assists in screening for comorbid anxiety presentations. The IUS and ASI-R act as mechanistic predictors, capturing cognitive and interoceptive vulnerability dimensions that contribute to anxiety maintenance.

Neurocognitive functioning will be evaluated using the CNS Vital Signs battery, while stress exposure will be assessed using the Stress and Adversity Inventory. Affective states will be measured using the Positive and Negative Affect Schedule (PANAS), which assesses momentary affective experiences across two dimensions: positive and negative affect. The Behavioral Inhibition and Activation Systems (BIS/BAS) scales will be included to evaluate motivational and affect-regulatory tendencies. These measures contribute to the RDoC Arousal and Regulatory Systems domain, capturing interindividual variability in emotional and motivational responsiveness.

To reduce participant burden and ensure longitudinal feasibility, shortened versions of selected questionnaires will be administered at interim visits (2 and 12 months). Remote administration via telephone or a secure institutional online platform may be employed as appropriate. All remote procedures will use validated formats and standardized instructions to maintain reliability and data comparability across timepoints. Detailed descriptions of each instrument—including theoretical constructs, administration procedures, psychometric properties, and language validation—are provided in the Supplementary Material (Table S1).

#### 2.5.3. VR-Based Behavioral Assessment

A VR environment will be used to assess anxiety-related behavioral responses in an ecologically valid and controlled setting. The system integrates physiological, behavioral, and neural data streams during exposure to anxiety-inducing scenarios.

The VR assessment will span approximately 30 min, including a 20-min task conducted within a fully immersive environment. The platform is built on the previously validated Virtual Reality Assessment for Panic Disorder (VRA-PD), which originally comprised three modules: daily environment exposure (M1), relaxation (M2), and interoceptive exposure (M3) [25]. In the validation study by Kim et al., the VRA-PD successfully distinguished patients with panic disorder from healthy controls across multiple physiological and subjective indices [25]. In this protocol, the interoceptive exposure module (M3) was excluded to reduce participant burden and improve data quality. In the original VRA-PD feasibility study [25], M3-related data were partially excluded due to user discomfort and motion-related physiological artifacts. Consistent with these findings, recent VR-based predictive modeling studies also excluded M3 to minimize movement- and respiration-related noise. Accordingly, the structure and content of M1 and M2 have been retained.

Although initially designed for panic disorder, the paradigm targets fundamental threat-related processes—anticipatory anxiety, avoidance, and physiological arousal—that are shared across anxiety disorders. The M1 (daily environment exposure) module induces claustrophobic and social-evaluative anxiety by requiring participants to enter and remain in an elevator occupied by several virtual avatars, while the M2 (relaxation) module trains diaphragmatic breathing and progressive muscle relaxation to assess recovery dynamics. This structure enables examination of both anxiety induction and regulation within ecologically valid environments. Stimuli are presented through an immersive head-mounted display (Oculus Quest Pro; Meta Platforms, Menlo Park, CA) with a native resolution of 1800 × 1920 pixels per eye, 90 Hz refresh rate, and a 106° field of view. Built-in off-ear speakers deliver synchronized auditory cues to enhance realism and immersion. The system supports 6-degree-of-freedom (6DoF) tracking and built-in eye and facial-expression tracking, which enable the recording of gaze fixation and subtle behavioral responses. Each module lasts approximately 10 min, and the total VR session duration is about 25 min.

All stimuli are implemented in Unity 3D 2022, with standardized lighting, ambient sound, avatar animation, and environmental layout to ensure reproducibility across participants and sessions. Task scripts automatically control scenario timing, transitions, and data logging, allowing identical exposure parameters for all participants.

Behavioral metrics such as task completion time, in-environment decisions, and subjective responses will be collected throughout the task. These data, alongside physiological signals, allow for high-resolution multimodal characterization of individual anxiety responses.

This refined VR system enables repeated and scalable deployment in both clinical and research settings. It is conceptually aligned with the Potential Threat construct in the Negative Valence Systems domain of the RDoC framework.

Task delivery scripts, environmental parameters, and timing are fully automated to ensure identical exposure across participants and sessions. All raw and event-timed data are timestamp-synchronized and stored for reproducibility. Prior testing confirmed high within-session reliability of anxiety ratings and heart rate variability (HRV) measures [25]. Through this adaptation, the VR paradigm provides a standardized yet ecologically rich framework for probing shared mechanisms of anxiety beyond diagnostic boundaries.

#### 2.5.4. EEG and ECG Acquisition

Continuous electrophysiological activity will be acquired using a 20-channel wireless EEG system (Enobio; Neuroelectrics, Barcelona, Spain), which allows simultaneous EEG and ECG acquisition within the VR environment. Nineteen scalp electrodes will be positioned according to the international 10–20 system, and one additional channel will record a single-lead ECG from the chest. All sensors use Ag/AgCl electrodes with an impedance kept below 10 kΩ. Data will be sampled at 500 Hz (24-bit resolution) with a 0.1–100 Hz online band-pass filter and referenced to the common-average reference.

EEG/ECG streams are hardware-synchronized with VR event triggers and behavioral data through a shared digital timestamp system, allowing precise temporal alignment across modalities. This configuration supports combined analyses linking neural oscillatory dynamics and autonomic responses to VR-elicited anxiety behavior.

#### 2.5.5. Behavioral and Postural Tracking

Upper-body posture and movement during the VR tasks will be continuously captured using the AVATAR Studio system [29]. The original AVATAR platform reconstructs 3D body motion in real time using a multi-camera, markerless tracking architecture and deep-learning-based body-part detection (AVATARnet), enabling high-resolution behavioral quantification in mice.

In the present study, the system will be configured for human participants using four infrared cameras positioned to ensure full-angle visibility and minimal occlusion. The resulting three-dimensional skeletal joint trajectories (60 Hz) will be treated as four-dimensional spatiotemporal data (x, y, z × time) and analyzed using unsupervised learning approaches to derive latent movement representations. These data-driven behavioral representations will be used to characterize patterns such as postural freezing, avoidance behavior, and upper-body displacement during VR tasks. Derived motion features will be synchronized with EEG, ECG, and VR event data for multimodal integration. Depending on the analytic aims, both kinematic and temporal–dynamical models (e.g., movement entropy, transition-state clustering) may be applied to characterize behavioral responses.

#### 2.5.6. Brain MRI Acquisition

Brain MRI will be acquired using clinical-grade MRI equipment (A 3 T Philips Ingenia scanner, Philips Healthcare, Best, The Netherlands) at the Yonsei Convergence Medical Technology Center (Seoul, Republic of Korea). The proposed MRI machine is a 3.0-T Philips Ingenia CX (Philips Healthcare, Best, The Netherlands). The scan will take approximately 40 min, capturing T1 3D coronal structural brain images, resting-state functional brain images (fMRI), and diffusion-weighted images (DWI). MRI acquisition will follow a standardized protocol designed to assess cortical structure, intrinsic functional connectivity, and white matter integrity.

Structural imaging will be acquired using a 3D T1-weighted magnetization-prepared rapid gradient echo sequence to assess cortical thickness and gray matter volume. Resting-state fMRI will be collected with participants instructed to remain still with eyes closed and to avoid focused thinking, enabling assessment of intrinsic functional connectivity. DWI will be used to evaluate white matter integrity through metrics such as fractional anisotropy and mean diffusivity.

Participants will wear earplugs and headphones for acoustic protection and will be stabilized using foam pads to minimize motion artifacts. MRI contraindications (e.g., metallic implants, pregnancy) will be screened prior to scanning. A trained research assistant will be present throughout to monitor for distress or discomfort.

### 2.6. Outcomes and Operational Definitions

The primary outcome of this study is treatment response, operationally defined as a clinically meaningful reduction in panic symptom severity. Responders are defined as participants who achieve a ≥40% decrease in the total score of the PDSS from baseline to each follow-up assessment (2, 6, and 12 months), while participants not meeting this criterion are classified as non-responders.

This threshold was selected based on prior evidence indicating that a 35–45% decrease on disorder-specific anxiety scales corresponds to clinically meaningful improvement and early treatment benefit [30,31]. The 40% criterion also provides adequate sensitivity for detecting within-subject improvement across multiple timepoints in longitudinal designs.

To ensure conceptual coherence with the study’s transdiagnostic framework, additional analyses will examine changes in the GAD-7, DASS-21 Anxiety subscale, and LSAS as secondary outcomes, and the consistency of responder classification across these measures will be evaluated. This multi-metric approach retains sensitivity to panic-specific reactivity while enabling comparability with broader anxiety-disorder literature.

These operational definitions allow for both categorical classification (responder vs. non-responder) and continuous modeling of symptom change, facilitating the integration of clinical, behavioral, physiological, and neuroimaging predictors across the 12-month follow-up.

### 2.7. Sample Size and Power Considerations

This study is designed to identify predictive biomarkers of treatment response through multimodal data modeling in a prospective, observational framework. Since the primary goal is to develop prediction models rather than to test predefined hypotheses, traditional power calculations based on inferential statistics are not directly applicable.

For the cross-sectional comparison between the clinical anxiety group and the healthy control group, a medium effect size (Cohen’s d = 0.5) was assumed based on prior neuroimaging meta-analyses [32]. This yielded a minimum required sample size of 36 participants per group to achieve 80% power at a two-sided α = 0.05. Accounting for a 10% attrition rate, the control group target was set at 40 participants.

Sample size planning for predictive modeling was based on guidelines by Riley et al. [33], which recommend ensuring a sufficient number of events per predictor parameter to reduce the risk of model overfitting. Although the dataset includes numerous raw variables across clinical, behavioral, physiological, and neuroimaging domains, only a subset (~20 variables) will be used in each model following feature selection procedures. Assuming a non-response rate of 25% (the reported remission rate of 38–75% at 6 months) [34,35], at least 80 non-responders would be required to ensure four events per predictor. Accordingly, the clinical anxiety group was targeted at 145 participants to allow for a 20% dropout rate and ensure sufficient statistical power for model development.

## 3. Data Analysis and Statistical Methods

### 3.1. EEG and ECG Preprocessing and Feature Extraction

EEG preprocessing will follow the principles proposed by Delorme [36], emphasizing minimal and transparent signal manipulation to preserve neural signal integrity. A single 0.5-Hz high-pass filter will be applied at data loading to prevent distortion of independent component analysis (ICA) outcomes. Automated artifact cleaning (clean_rawdata [37]) will identify and interpolate flat or low-correlation channels, and ICA (runica, maxsteps = 500, double precision) will be performed for source separation. Independent components will be classified using ICLabel [38], and non-brain components with ≥0.90 confidence will be removed. This preprocessing strategy—derived primarily from Delorme [36]—is expected to enhance reproducibility, minimize preprocessing bias, and maintain the natural covariance structure of the EEG. Additional spectral or connectivity analyses (e.g., Welch power spectra, coherence, or source-level metrics) may be applied depending on the analytic objectives.

For ECG, R-peaks will be detected using established algorithms such as the Pan–Tompkins algorithm [39] or more recent approaches including adaptive methods for wearable sensors [40] and deep learning-based techniques utilizing U-Net and LSTM architectures [41,42,43], depending on signal quality and computational requirements. Physiologically implausible beats will be excluded through automated quality assessment prior to computing HRV indices.

For spectral analysis of HRV, multiple approaches will be considered, including the traditional Welch’s method [44]; the multitaper method, which offers superior variance-bias trade-offs [45,46]; and adaptive multitaper techniques optimized for biological signals [47,48]. Standard HRV parameters (standard deviation of NN interval, root mean square of successive differences, low frequency, high frequency, low-to-high frequency ratio) will be computed, and may be complemented by non-linear dynamics measures (e.g., sample entropy, detrended fluctuation analysis), time–frequency analyses (e.g., wavelet transform, Hilbert–Huang transform), and complexity metrics in exploratory analyses to comprehensively characterize autonomic nervous system dynamics.

All EEG/ECG features will be time-locked to VR events and behavioral indices(three-dimensional positional information of skeletal-points extracted from AVATAR Studio) to enable multimodal integration with clinical and neuroimaging data.

### 3.2. Brain MRI Analysis

Preprocessing for MRI data will use standardized pipelines: fMRIPrep v24.0 for T1 and resting-state fMRI (anatomical segmentation, motion correction, spatial normalization, and smoothing at 6 mm full-width half maximum), and QSIPrep v0.21 for DWI (eddy-current correction, bias-field correction, and tensor fitting).

Structural MRI, diffusion-weighted imaging (DWI), and resting-state fMRI will be analyzed to comprehensively characterize brain structure, white matter integrity, and intrinsic functional organization associated with anxiety and treatment response. Structural MRI will yield cortical thickness and gray matter volume measures; DWI will provide white matter microstructural indices, including fractional anisotropy and mean diffusivity; and resting-state fMRI will be used to derive functional connectivity, amplitude of low-frequency fluctuations, and regional homogeneity as candidate neurobiological features for group comparisons and predictive modeling.

Group-level comparisons will be performed between (i) healthy controls and anxiety participants and (ii) responders and non-responders using general linear models with age and sex as covariates. Multiple comparisons will be adjusted using the false discovery rate. Significant neural indices will be further examined as candidate predictive features in multimodal modeling.

### 3.3. Predictive Modeling of Treatment Response

The primary objective is to develop machine-learning models that predict treatment response (≥40% PDSS reduction at 2, 6, and 12 months). Binary (responder vs. non-responder) and three-group models (healthy control, responder, non-responder) will be evaluated. Predictor features will include sociodemographic and psychiatric-history variables, clinical and psychometric scores, VR-derived behavioral indices, EEG/ECG features, 3D skeletal tracing, and neuroimaging markers. In most models, age and sex will be treated as basic covariates given their known influence on neural and behavioral measures. Lifestyle variables (e.g., education, smoking, alcohol use) and treatment-related clinical variables (medication use and psychotherapy) will be systematically recorded and incorporated either as covariates to adjust for treatment effects or as candidate predictive features, depending on the specific modeling objective and analytic context.

Predictor inclusion and feature reduction will be guided by a combination of theoretical relevance and empirical criteria. Candidate variables will undergo an initial screening based on prior literature and clinical interpretability. Dimensionality will then be reduced using data-driven feature-selection procedures, such as penalized regression methods (e.g., LASSO) or feature-importance-based ranking, with the number of retained features determined by model performance and stability rather than a predefined target. Variables exhibiting substantial multicollinearity (e.g., variance inflation factor > 5), redundancy, or consistently low contribution across cross-validation folds may be excluded prior to model fitting.

All features will be standardized, and missing values handled using multiple imputations by chained equations or similar robust approaches. Depending on data characteristics, dimensionality-reduction techniques (e.g., principal component analysis) may be applied before model fitting.

**Figure 3:**
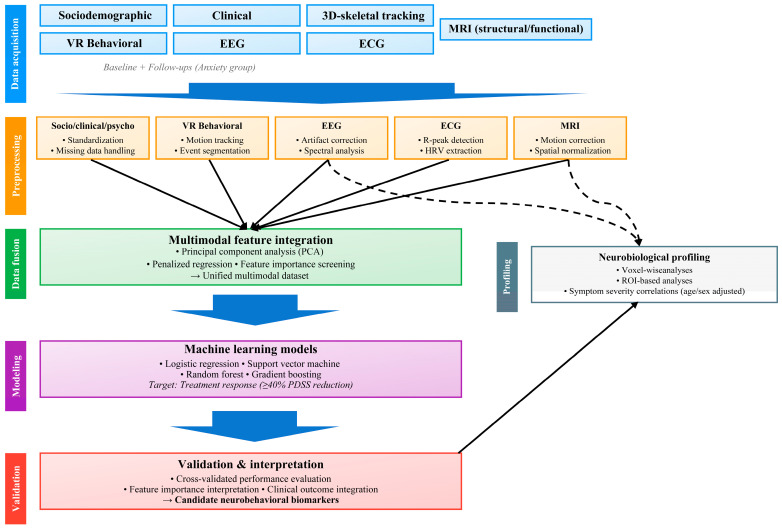
Analytical framework for multimodal treatment response prediction in patients with anxiety disorder. The six-stage pipeline progresses from multimodal data acquisition (sociodemographic, clinical, psychometric, VR-behavioral, neurophysiological, and neuroimaging) through standardized preprocessing, feature integration via dimensionality reduction, machine learning-based prediction (≥40% PDSS reduction), parallel neurobiological profiling, and validation to identify neurobehavioral biomarkers. EEG and MRI data undergo both integrated analysis and direct profiling (dashed arrows).

The full multimodal analytic workflow supporting these predictive models is summarized in Figure 3. This figure outlines the end-to-end structure of the pipeline, beginning with modality-specific preprocessing (sociodemographic/clinical variables, VR behavioral data, EEG/ECG signals, 3D-skeletal tracking, and MRI), followed by a multimodal feature-integration stage, and culminating in supervised-learning models and validation procedures. This framework visually captures how heterogeneous data streams are harmonized into a unified dataset for treatment-response prediction and how complementary neurobiological profiling analyses interface with the main machine-learning pipeline.

Various supervised-learning algorithms—such as logistic regression, support-vector machines, random forests, or gradient-boosting methods (e.g., XGBoost, CatBoost)—may be compared. Model performance will typically be evaluated through k-fold or nested cross-validation, using metrics such as area under the curve, F1 score, and balanced accuracy. Model interpretability will be explored through feature-importance or SHapley Additive exPlanations-value analyses. All analytic pipelines will be version-controlled to ensure reproducibility.

### 3.4. Statistical Considerations

All analyses will be exploratory and hypothesis-generating. Normality of data distributions will be verified using the Shapiro–Wilk test and residual diagnostics. If assumptions for parametric tests are violated, appropriate transformations or non-parametric alternatives will be employed. Potential covariates (age, sex, medication status, treatment type) will be included as needed. Missing data will be handled using multiple imputation or complete-case analysis, depending on the extent and mechanism of missingness.

To ensure generalizability, model interpretability, and prevention of overfitting, we will report confidence intervals, cross-validation performance variability, and model calibration statistics where applicable. Robustness checks (e.g., bootstrapping with 1000 iterations) will be performed to confirm model stability. All analyses will be implemented in R (version 4.3.1 or later) and Python (version 3.10 or later). The analysis code will be managed using a version-controlled repository (Git), and the final software environment, including package versions, will be documented to ensure reproducibility.

## 4. Benefits and Risks to Participants

This study involves minimal risk, as it is based on non-invasive and observational procedures. However, certain assessments may cause temporary discomfort or psychological distress. Participants may experience mild anxiety during clinical interviews or VR-based behavioral tasks that simulate stress-inducing situations. Additional discomfort may arise from the application of EEG/ECG sensors, potential motion sickness during VR exposure, or feelings of claustrophobia during MRI scanning.

To mitigate these risks, participants will be fully informed in advance about all procedures and potential discomforts. They will be allowed to take breaks or discontinue specific assessments at any point. A trained clinician or research staff member will be present throughout all study procedures to monitor participant well-being and respond promptly if distress arises.

Participation in this study may provide benefits such as access to a comprehensive psychiatric evaluation and enhanced insight into one’s psychological health. Participants in the clinical anxiety group will also receive longitudinal follow-up, which may contribute to self-monitoring and inform future clinical care. While no therapeutic intervention is provided within the research protocol, information gathered during the study may support personalized treatment planning.

Participation is entirely voluntary. Individuals may withdraw at any time without consequence to their current or future medical care. If needed, participants will be referred for appropriate clinical services.

## 5. Ethical Approval

This study protocol has been reviewed and approved by the Institutional Review Board (IRB) of Samsung Kangbuk Hospital (IRB number: 2024-10-018-002) in accordance with the Declaration of Helsinki (2013 revision) and International Council for Harmonization Good Clinical Practice guidelines. The IRB reviewed the study protocol, informed consent forms, recruitment materials, and all associated documentation to ensure the protection of participant rights, safety, and well-being.

Written informed consent will be obtained from all participants before study enrollment. The consent process will include comprehensive information about the study’s aims, procedures, potential risks and benefits, confidentiality protections, and the right to withdraw without penalty.

Participants will be provided with the contact information of the principal investigator and designated research personnel, enabling them to report concerns, adverse events, or protocol-related inquiries. In the event of serious adverse reactions, appropriate clinical support will be provided, and the IRB will be notified as required.

## 6. Management and Protection of Personal Information

All data collected in this study—including clinical information, questionnaire responses, physiological signals (e.g., EEG, ECG), behavioral performance data from the VR task, and brain imaging data—will be managed according to institutional, national, and international guidelines for research data protection.

Each participant will be assigned a unique study-specific identification code at the time of enrollment. This code will be used to label all datasets and files. Personally identifiable information (e.g., name, date of birth, contact information) will be stored separately from research data in a secure, access-restricted location and will be linked to study data only via the assigned code.

All electronic data will be stored on encrypted and password-protected systems, with access limited to only authorized research personnel. Hardcopy documents (e.g., signed consent forms and screening logs) will be stored in locked cabinets in secured research offices.

According to the Bioethics and Safety Act and institutional policies, research data will be retained for at least 3 years following the completion of the study. After this retention period, data will be disposed of securely using digital deletion and physical document shredding protocols. Regular internal audits will be conducted to ensure compliance with data protection standards, including review of informed consent forms, data files, and access logs.

## 7. Discussion

This protocol outlines a multidimensional, prospective investigation designed to improve the assessment and prediction of anxiety-related psychopathology through the integration of behavioral, physiological, and neuroimaging data. Grounded in the RDoC framework, the study aims to move beyond symptom-based diagnostic systems by identifying neurobiologically informed and transdiagnostic markers of treatment response.

Methodologically, the study introduces several innovations. These include a VR-based behavioral assessment platform that enables standardized induction of anxiety responses in immersive scenarios, concurrent recording of EEG and ECG signals, and real-time 3D skeletal tracking. By incorporating these multimodal data streams, the study allows for high-resolution behavioral and physiological characterization of individual anxiety responses.

The combination of VR-derived features with structural and functional neuroimaging facilitates the development of predictive models using machine learning techniques. This approach supports early identification of treatment response trajectories and the possibility of stratifying individuals based on underlying neurobiological patterns—core goals of precision psychiatry.

This study provides a feasible and scalable alternative to traditional RDoC research designs, which have often faced implementation barriers such as a lack of standardized recruitment criteria and reliance on resource-intensive, expert-administered assessments [13,49]. Recent trends indicate a shift toward transdiagnostic, symptom-based recruitment and the incorporation of cost-efficient digital tools to operationalize RDoC constructs in real-world settings [50]. In line with this evolution, our study embeds RDoC principles within a clinically grounded, transdiagnostic framework using widely accessible assessments. By integrating VR-based behavioral tasks, physiological monitoring, and neuroimaging, this design enables dimensional characterization of anxiety in a manner that is both mechanistically rich and practically scalable. As such, it bridges the gap between theoretical innovation and clinical applicability, advancing the translational potential of RDoC-informed research.

This study has several methodological and practical limitations. First, the longitudinal design is asymmetric: healthy controls are assessed twice (baseline and 12 months), whereas the anxiety group is followed three times (2, 6, and 12 months), limiting short-term trajectory comparisons. Second, ongoing medication and psychotherapy may confound treatment-response estimates, although these variables will be recorded and statistically adjusted. Third, combining multimodal data (VR, EEG, ECG, MRI) entails potential synchronization errors, variable signal quality, and data-loss risks. Standardized preprocessing and quality-control pipelines have been implemented, but residual heterogeneity may remain. Finally, practical issues such as participant burden, VR sensitivity, and attrition could affect data completeness and generalizability. Despite these constraints, the protocol emphasizes reproducibility and transparent analytic procedures to support robust, hypothesis-generating findings on neurobehavioral predictors of treatment response. In summary, this protocol outlines a longitudinal, multimodal investigation designed to identify neurobehavioral predictors of treatment response in individuals with anxiety disorders. By aligning clearly defined outcomes with standardized acquisition and analysis pipelines across behavioral, physiological, and neuroimaging domains, the study aims to generate reproducible, mechanistic insights into the processes underlying anxiety improvement. The inclusion of harmonized procedures, cross-modal synchronization, and transparent analytic workflow diagrams ensures methodological rigor and clarity, thereby facilitating future replication and data sharing within the field.

## Data Availability

Not applicable.

## Supplementary Materials

The following supporting information can be downloaded at: https://www.mdpi.com/article/10.3390/jcm15010007/s1, Table S1: ASSESSMENT TOOLS AND THEORETICAL CONSTRUCTS. References [51,52,53,54,55,56,57,58,59,60,61,62,63,64,65,66,67,68,69,70,71,72,73,74,75,76,77,78] are cited in the Supplementary Materials.

## Abbreviations

The following abbreviations are used in this manuscript:

ASI-R	Anxiety Sensitivity Index-Revised
BIS/BAS	Behavioral Inhibition/Activation System Scales
CNSVS	CNS Vital Signs Battery
DASS-21	Depression Anxiety Stress Scales-21
DSM	Diagnostic and Statistical Manual of Mental Disorders
DWI	Diffusion-weighted imaging
ECG	Electrocardiography
EEG	Electroencephalography
fMRI	Functional magnetic resonance imaging
GAD	Generalized anxiety disorder
GAD-7	7-item Generalized Anxiety Disorder Scale
HRV	Heart rate variability
ICA	Independent component analysis
IUS	Intolerance of Uncertainty Scale
LSAS	Liebowitz Social Anxiety Scale
MINI	Mini International Neuropsychiatric Interview
MRI	Magnetic resonance imaging
PANAS	Positive and Negative Affect Schedule
PDSS	Panic Disorder Severity Scale
RDoC	Research Domain Criteria
SAD	Social anxiety disorder
SSQ	Simulator Sickness Questionnaire
STRAIN	Stress and Adversity Inventory
VR	Virtual reality
