# Supplementary material for: Identifying Neurobehavioral Biomarkers of Anxiety and Treatment Response Using Virtual Reality, Electroencephalography, Magnetic Resonance Imaging, and Related Multimodal Assessments: A Longitudinal Study Protocol
Source: J Clin Med. 2025 Dec 19;15(1):7. doi: 10.3390/jcm15010007 (PMC12787110; doi:10.3390/jcm15010007)
Supplement: Supplementary file 1 [file jcm-15-00007-s001.zip › jcm-4030764-supplementary.pdf]

Supplementary Material

ASSESSMENT TOOLS AND THEORETICAL CONSTRUCTS

Several of the selected measures were chosen based on their alignment with constructs defined in the Research Domain Criteria (RDoC) framework. Specifically, the Intolerance of Uncertainty Scale (IUS) and the Anxiety Sensitivity Index (ASI) are associated with the *Potential Threat* and *Sustained Threat* constructs within the Negative Valence Systems domain. The Behavioral Inhibition and Activation Scales (BIS/BAS) correspond to *Approach Motivation* and *Inhibitory Control* within the Positive Valence Systems and Cognitive Systems domains. The Positive and Negative Affect Schedule (PANAS) reflects affective responsiveness under the Arousal/Regulatory Systems domain. The CNS Vital Signs Battery captures cognitive functioning relevant to the Cognitive Systems domain, such as working memory, attention, and executive function [51].

Panic Disorder Severity Scale (PDSS)

The Panic Disorder Severity Scale (PDSS) is a clinician-administered measure comprising seven items rated on a 5-point scale (0–4), assessing panic attack frequency, distress, anticipatory anxiety, avoidance, and functional impairment [52]. The total score ranges from 0 to 28, with higher scores indicating greater severity. The PDSS has strong reliability (Cronbach's α = 0.88–0.94) and validity, making it useful for symptom assessment and treatment monitoring. A Korean version of the PDSS (PDSS-K) has been developed and validated, showing good psychometric properties, including a high internal consistency (Cronbach's α = 0.91) and strong concordance with the original scale, supporting its applicability in Korean clinical and research settings [53].

Liebowitz Social Anxiety Scale (LSAS)

The Liebowitz Social Anxiety Scale (LSAS) is a 24-item self-report questionnaire designed to assess fear and avoidance in social and performance situations for diagnosing social anxiety disorder. Each item is rated on a 4-point Likert scale (0–3), with total scores ranging from 0 to 144, where higher scores indicate greater severity [54]. The LSAS is widely used in clinical research to evaluate treatment outcomes, particularly in pharmacological and cognitive-behavioral therapy studies. It has demonstrated strong reliability (Cronbach's α = 0.89–0.94) and validity, supporting its use in both clinical and research settings [55]. A Korean version with good internal consistency (Cronbach's α = 0.93 [fear], 0.90 [avoidance]) and suitability for Korean-speaking populations has been developed and validated [56].

7-item Generalized Anxiety Disorder Scale (GAD-7)

The Generalized Anxiety Disorder-7 (GAD-7) is a widely used self-report screening tool designed to assess the presence and severity of generalized anxiety disorder symptoms [57]. It comprises seven items rated on a 4-point Likert scale (0 = not at all to 3 = nearly every day), yielding a total score ranging from 0 to 21. Higher scores reflect greater levels of anxiety symptomatology. The Korean version of the GAD-7 has been developed and validated, showing good psychometric properties, including a high internal consistency (Cronbach's α = 0.92) and strong concordance with the original scale [58].

Depression, Anxiety, and Stress Scale (DASS)

The Depression, Anxiety, and Stress Scale-21 (DASS-21) is a self-report instrument developed by Antony et al. to assess symptoms of depression, anxiety, and stress across diverse populations [59]. In 2018, Lee et al. translated and validated the Korean version (K-DASS-21), ensuring linguistic and cultural appropriateness. Psychometric evaluation in a

sample of 481 Korean adults demonstrated high internal consistency, with a Cronbach's α of 0.93, supporting its reliability for clinical and research purposes [60].

Intolerance of Uncertainty Scale (IUS)

The Intolerance of Uncertainty Scale is a self-report measure assessing an individual's tendency to perceive uncertain or ambiguous situations as stressful or threatening [61]. It captures cognitive, emotional, and behavioral responses to uncertainty, which theoretically play a key role in the development and maintenance of anxiety disorders—particularly generalized anxiety disorder [62].

The version used in this study comprises 12 items rated on a 5-point Likert scale (1 = not at all characteristic of me to 5 = entirely characteristic of me), with higher scores indicating greater intolerance of uncertainty. The IUS aligns conceptually with the *Potential Threat* construct within the Negative Valence Systems domain of the RDoC framework. A Korean version has been developed and validated, showing good internal consistency (Cronbach's α = 0.89) and applicability in Korean-speaking populations [63].

Anxiety Sensitivity Index-Revised (ASI-R)

The Anxiety Sensitivity Index-Revised (ASI-R) is a 36-item self-report instrument designed to assess anxiety sensitivity, which refers to the fear of anxiety-related sensations based on beliefs that these sensations may result in harmful physical, psychological, or social consequences. Each item is rated on a 5-point Likert scale (0 = very little to 4 = very much), with higher scores indicating greater anxiety sensitivity. The ASI-R expands upon the original ASI by incorporating a multidimensional structure, assessing physical, cognitive, and social concerns associated with anxiety sensitivity [64]. It has been widely used to identify vulnerability factors for the development and maintenance of anxiety disorders and is

considered particularly relevant to panic disorder and generalized anxiety disorder [65]. A Korean version of the ASI-R (K-ASI-R) has been developed and validated, showing high internal consistency (Cronbach's α = 0.93) and supporting its applicability in Korean-speaking populations [66]. Within the RDoC framework, anxiety sensitivity maps onto constructs such as *Potential Threat* and *Sustained Threat* in the Negative Valence Systems domain.

Behavioral Inhibition and Activation System (BIS/BAS)

The Behavioral Inhibition and Activation System (BIS/BAS) scales assess individual differences in sensitivity to punishment (Behavioral Inhibition System, BIS) and reward (Behavioral Activation System, BAS), respectively, based on Gray's reinforcement sensitivity theory [67]. The BIS measures responsiveness to aversive stimuli and the tendency toward anxiety, while the BAS evaluates approach behavior toward rewarding stimuli [68].

The BIS scale comprises seven items, while the BAS scale includes 13 items divided into three subscales: reward responsiveness, drive, and fun-seeking. Higher BIS scores reflect a greater tendency toward anxiety and avoidance-based behavior in response to potential punishment or negative outcomes. Higher BAS scores indicate increased motivation to pursue rewarding experiences. In the original study, the BIS scale demonstrated a Cronbach's α of 0.74, indicating acceptable internal consistency. The BAS scale was divided into three subscales: Reward Responsiveness (α = 0.73), Drive (α = 0.76), and Fun Seeking (α = 0.66) [69]. The Korean version of the BIS/BAS scales has demonstrated strong psychometric properties, with internal consistency coefficients of α = 0.833 for BIS and α = 0.831 for BAS, supporting their applicability in Korean clinical and research contexts [70].

Within the RDoC framework, the BIS is conceptually linked to the *Potential Threat* construct in the Negative Valence Systems domain, while the BAS relates to *Approach Motivation* and *Reward Learning* within the Positive Valence Systems domain.

Positive and Negative Affect Schedule (PANAS)

The Positive and Negative Affect Schedule (PANAS) is a 20-item self-report measure designed to assess two independent dimensions of affect: Positive Affect (PA) and Negative Affect (NA). It comprises 20 items, with 10 items measuring PA and 10 items measuring NA. Each subscale comprises 10 items rated on a 5-point Likert scale (1 = very slightly or not at all to 5 = extremely), yielding scores that range from 10 to 50 per dimension. Higher scores indicate greater intensity or frequency of the corresponding affective state [71].

In the original study, both scales demonstrated high internal consistency, with Cronbach's α coefficients ranging from 0.86−0.90 for PA and 0.84−0.87 for NA [72]. A Korean version (K-PANAS) has been developed and validated, showing good reliability and validity, with Cronbach's α of 0.87 for PA and 0.91 for NA [73].

The PANAS is widely used in both clinical and research contexts to assess baseline emotional states as well as changes in affect over time. It is also commonly employed in studies on mood disorders, stress response, and emotional regulation. Within the RDoC framework, the PANAS is aligned with constructs under the *Arousal and Regulatory Systems* domain, capturing individual differences in affective responsiveness.

CNS Vital Signs Battery

The CNS Vital Signs Battery is a computerized neurocognitive assessment battery developed to evaluate multiple domains of cognitive functioning, including memory, attention, and

executive control. It has been widely employed in both clinical and research settings for cognitive profiling, treatment response monitoring, and longitudinal follow-up.

In this study, a subset of the CNS Vital Signs battery will be administered, focusing on five specific domains: verbal memory, visual memory, Stroop test performance, shifting attention, and perception of emotions. These tasks were selected based on their relevance to cognitive processes commonly impaired in anxiety disorders and their alignment with constructs within the *Cognitive Systems* domain of the RDoC framework—such as *Working Memory*, *Attention*, *Cognitive Control*, and *Social Cognition*.

The CNS Vital Signs battery has demonstrated strong psychometric properties, with evidence supporting its test-retest reliability, construct validity, and sensitivity to clinical change [74]. The computerized format enables standardized administration and automated scoring, facilitating efficient data collection and reducing inter-rater variability.

Simulator Sickness Questionnaire (SSQ)

The Simulator Sickness Questionnaire (SSQ) is a self-report instrument developed to assess symptoms associated with simulator-induced motion sickness, particularly in VR and motion-based environments. It comprises 16 items rated on a 4-point Likert scale (0 = none to 3 = severe), covering three symptom clusters: nausea, oculomotor disturbances, and disorientation. A total severity score, as well as subscale scores, can be calculated.

The SSQ is widely used in studies involving VR environments to monitor participant discomfort and ensure the safety and interpretability of physiological and behavioral data [75]. It is especially useful for controlling confounding effects in experiments involving immersive technologies.

A Korean version of the SSQ (K-SSQ) has been developed and validated, showing good psychometric properties, including a Cronbach's α of 0.824, supporting its applicability in Korean VR-based research contexts [76].

Stress and Adversity Inventory (STRAIN)

The Stress and Adversity Inventory (STRAIN) is a computerized, structured interview system designed to assess cumulative exposure to life stressors across the lifespan. It evaluates both acute and chronic stressors in 14 psychosocial domains, including interpersonal, occupational, financial, and health-related contexts [77].

Participants will report on the frequency, severity, timing, and duration of each endorsed stressor, providing a comprehensive profile of their lifetime stress exposure. The STRAIN has previously demonstrated strong psychometric properties, including high test-retest reliability ($r = 0.90$), concurrent validity with related stress ($r = 0.55$, $p < 0.001$, with CTQ-SF), and predictive validity for recent mental health complaints ($\beta = 0.15$, Kessler's smitten psychological distress inventory) and physical health complaints ($\beta = 0.41$, Physical Health Questionnaire). A Korean version of STRAIN (K-STRAIN) has been developed and validated, showing good concurrent validity ($r = 0.61$, $p < 0.05$, with CTQ-SF), robust predictive validity for anxiety ($\beta = 0.17$, GAD-7) and depression ($\beta = 0.17$, Patient Health Questionaire-9), and high test-retest reliability ($r = 0.91$). The instrument is widely used in clinical and epidemiological research to examine the long-term impact of psychosocial stress [78].
